# Spatial Characterization of Landscapes through Multifractal Analysis of DEM

**DOI:** 10.1155/2014/563038

**Published:** 2014-08-06

**Authors:** P. L. Aguado, J. P. Del Monte, R. Moratiel, A. M. Tarquis

**Affiliations:** ^1^Departamento de Producción Vegetal, Botánica, E.T.S.I.A., UPM, 28040 Madrid, Spain; ^2^Departamento de Producción Vegetal, Fitotecnia, E.T.S.I.A., UPM, 28040 Madrid, Spain; ^3^CEIGRAM, E.T.S.I.A., UPM, 28040 Madrid, Spain; ^4^Departamento de Matemática Aplicada, E.T.S.I.A., UPM, 28040 Madrid, Spain

## Abstract

Landscape evolution is driven by abiotic, biotic, and anthropic factors. The interactions among these factors and their influence at different scales create a complex dynamic. Landscapes have been shown to exhibit numerous scaling laws, from Horton's laws to more sophisticated scaling of heights in topography and river network topology. This scaling and multiscaling analysis has the potential to characterise the landscape in terms of the statistical signature of the measure selected. The study zone is a matrix obtained from a digital elevation model (DEM) (map 10 × 10 m, and height 1 m) that corresponds to homogeneous region with respect to soil characteristics and climatology known as “*Monte El Pardo*” although the water level of a reservoir and the topography play a main role on its organization and evolution. We have investigated whether the multifractal analysis of a DEM shows common features that can be used to reveal the underlying patterns and information associated with the landscape of the DEM mapping and studied the influence of the water level of the reservoir on the applied analysis. The results show that the use of the multifractal approach with mean absolute gradient data is a useful tool for analysing the topography represented by the DEM.

## 1. Introduction

Each landscape unit is defined by primary physiographic characteristics. In the landscape, several abiotic and biotic factors, as well as anthropic factors, interact to generate a characteristic dynamic over time. The focus of this study is an alluvial surface of arkose resulting from the erosion of the granite of the Sierra del Guadarrama produced by the factors cited above. These factors, along with their interactions at different scales, produce a strong modelling effect through erosion. The universal equation of hydraulic erosion presented by Wischmeyer and Smith (1978) [1] can be used to evaluate the intensity of this process. This model incorporates abiotic factors such as soil type, soil erodibility as a function of composition and structure, topographic factors described by the slope and its length, rain erosivity as function of rain volume, and precipitation intensity. In addition, the vegetation cover produces a biotic effect. In certain cases, anthropic factors, such as soil management and conservation, dominate the evolution of the landscape.

A digital elevation model (DEM) provides the information basis used for many geographic applications, for example, topographic studies, geomorphologic studies, and landscape analysis with geographic information systems (GIS). The ability of a DEM to represent the earth's surface depends on the surface roughness and the resolution used [2, 3]. The information in each DEM pixel depends on the scale used and is characterised by two variables, the resolution and the extension of the area studied [4]. DEMs can vary in resolution and accuracy according to the method used to produce the model [5, 6], although there are statistical characteristics that remain constant or highly similar over a broad range of scales [7]. Based on this property, several techniques have been applied to characterise DEMs through multiscale analysis [8] directly related to fractal geometry. In this way, the complexity of natural landscapes can be revealed [9, 10].

In the general mathematical framework of fractal geometry, many analytical methods have been developed. For example, textural homogeneity has been characterised using the fractal dimension [11]. The fractal dimension has also been used as a spatial measure for describing the complexity of remote sensing imagery [12]. Changes in image complexity have been detected through the spectral range of hyperspectral images affecting the fractal dimension [13], dependence of fractal dimension on the spectral bands of Landsat TM imagery De Cola [14], Lam [15], and other authors [16]. The use of multifractal/wavelet techniques is becoming more widespread in the analysis of remote sensing images [2, 17]; it is not as popular in DEM analysis, although there are several studies characterising soil surface microrelief [18].

Motivated by the fractal geometry of sets [19, 20], the development of multifractal (MF) theory, introduced in the context of turbulence, has been applied in many areas such as earthquake distribution analysis [21], soil pore characterisation [22, 23], image analysis [24], and remote sensing [25–35]. Research into relationships between landscape pattern and process has been influenced by the introduction of fractal geometry and the advent of fractal analysis [36]. With the increasing availability of high-resolution digital elevation data from increasingly larger areas, together with advances in geocomputation and geomorphometry, fractals have become of increasing interest for local-level environmental applications [37].

The acquisition of remotely sensed multiple spectral images is thus a unique source of data for determining the scale-invariant characteristics of the radiant fields related to many factors such as the chemical composition of soil and bedrock, their moisture content, and their surface temperature [28–30, 38–42]. In the MF scheme used, the digital elevation data are considered to represent a singular measure. The analysis then proceeds through an MF spectrum, which gives either geometrical or probabilistic information about the height distribution having the same singularity. Gagnon et al. [43] demonstrated on purely statistical grounds that monofractals are not sufficient to describe topography and that multifractals are needed. A profound review on how this topic has advanced can be found in Gagnon et al. [44].

There are scientific debates over what is fractal. However, a surface does not need to be multifractal to admit a multifractal analysis (MFA). The most important issues are whether MFA is a reliable method for determining fractal parameters and how the results of the MFA are to be interpreted in a given context [35]. We want to remark that the approach does not depend on the assumption that topography is fractal. This observation leads us to the general aim of the paper, which is to use MFA to characterise the information contained in DEM based on the original elevation data and on the absolute gradient. At the same time, we have investigated how the map information is affected by analysing the area under differing conditions, that is, for various water levels in the reservoir.

## 2. Materials and Methods

### 2.1. Site Description

**Figure 1:**
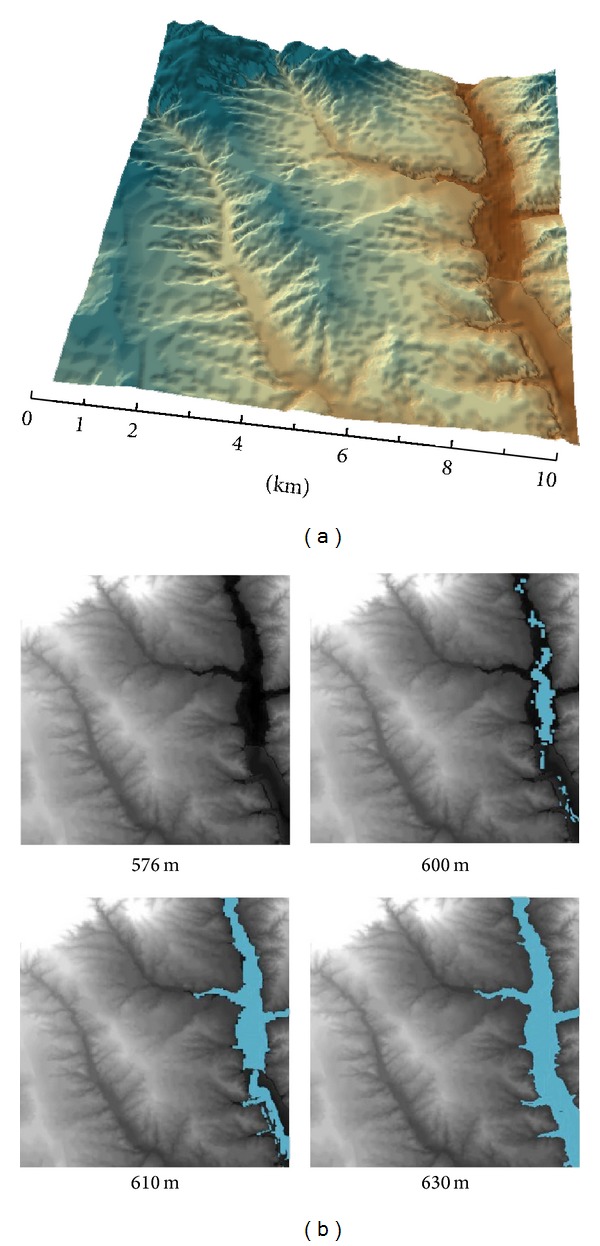
Visualization of DEM (1024 × 1024 data points) at the area studied (a) and the localization of the reservoir in the map at different filling levels (b) from emptiness (576 m) to full capacity (630 m).

The study area is represented by a 1024 × 1024 data matrix obtained from a DEM with a resolution of 10 × 10 m at each point and a height resolution of 1 m, which correspond with a region known as “*Monte de El Pardo*” a property of Spanish national heritage (*patrimonio nacional Español*) of 15,820 Ha located at a short distance from Madrid city with altitude ranging from 576 to 900 m and UTM coordinates Huse 30, Hemisphere Northern, *X*: 444312.312 to 434542.312 and *Y*: 4494542.408 to 4484312.408.* Manzanares* River goes through this area from north to south as it can be observed in Figure 1(a). In the southern area, a reservoir is found with a capacity of 43 hm^3^, with an altitude ranging from 576 m to 632 m when it is at the highest capacity as it is represented in Figure 1(b). In the middle of the reservoir, the minimum altitude of this area is achieved. Geologic characteristics of the area correspond to arkose deposits coming from granite and gneisses erosion, basis of the* Sierra de Guadarrama*. Several smooth slopes and a river network very few branched can be found with a surface ravaging. The potential vegetation is mainly of a Mediterranean occidental forest;* Q. ilex* L. is the climax specie with several shrub heliophilous vegetation and herbaceous (Gen.* Cistus*). There are some* Q. suber* L. isolated. Actually, this forest has been kept for hunting use.

The criteria of the selection of the study area were to delimit a homogeneous area with respect to soil characteristics and climatology, and then the topographic factor acquires a main role. Regarding vegetation cover, Mediterranean forest is present with some areas influenced by pasture characteristics as a consequence of historical use for hunting and a minimum soil management. With regard to anthropic factors, these have been much less than in the surrounding areas which have been cultivated, producing a high reduction in the original trees and shrubs of the area. However, in 1973, the construction of the reservoir on Manzanares River modified the water level equilibrium of some local streams at the same time than the main river in this area. A direct consequence was an alteration of the dynamic processes that shape this landscape.

### 2.2. Multifractal DEM Analysis

A multifractal analysis is basically the measurement of a statistic distribution and therefore gives useful information on a self-similar behaviour [45].

A monofractal object can be measured by counting the number *N* of *δ* size boxes needed to cover the object. The measure depends on the box size as

(1) $$\begin{array}{c} N\left(\delta \right)\propto \delta^{-D_{0}}, \end{array}$$

where

(2) $$\begin{array}{c} D_{0}=\underset{\delta \to 0}{\lim}\frac{\log N\left(\delta \right)}{\log\left(1/\delta \right)} \end{array}$$

is the fractal dimension. *D*_0_ is calculated from slope of a log-log plot.

There are several methods for implementing multifractal analysis; in this section, the selected moment method is explained [46]. This method uses mainly three functions: *τ*(*q*), known as the mass exponent function, *α*, the coarse Hölder exponent, and *f*(*α*), multifractal spectrum. A measure (or field), defined in two-dimensional data grid embedding space (*n* × *n* data points) and with values based on altitude (from 576 till 900 meters in this case), cannot be considered as a geometrical set and therefore cannot be characterized by a single fractal dimension.

Applying a nonoverlapping covering by boxes in an “up-scaling” partitioning process, we obtain the partition function *χ*(*q*, *δ*)  [47] defined as

(3) $$\begin{array}{c} \chi \left(q,\delta \right)=\overset{N(\delta )}{\underset{i=1}{\sum }}\mu_{i}^{q}\left(\delta \right)=\overset{N(\delta )}{\underset{i=1}{\sum }}m_{i}^{q}, \end{array}$$

where *m* is the mass of the measure, *q* is the statistical moments order, *δ* is the length size of the box, and *N*(*δ*) is the number of boxes in which *m*_*i*_ > 0. Based on this, the mass exponent function (*τ*(*q*)) shows the moments of the measure scales with the box size,

(4) $$\begin{array}{c} \tau \left(q\right)=\underset{\delta \to 0}{\lim}\frac{\log\langle \chi \left(q,\delta \right)\rangle }{\log\left(\delta \right)}=\underset{\delta \to 0}{\lim}\frac{\log\langle \sum_{i=1}^{N(\delta )}m_{i}^{q}\rangle }{\log\left(\delta \right)}, \end{array}$$

where 〈 〉 represents statistical moment of the measure *μ*_*i*_(*δ*) defined on a group of nonoverlapping boxes of the same size partitioning the area studied. This method is known as the method of moments [48].

The singularity index (*α*) can be determined by the Legendre transformation of the *τ*(*q*) curve [46] as

(5) $$\begin{array}{c} \alpha \left(q\right)=\frac{d\tau \left(q\right)}{dq}. \end{array}$$

The number of cells of size *δ* with the same *α*, *N*_*α*_(*δ*), is related to the cell size as *N*_*α*_(*δ*) ∝ *δ* ^−*f*(*α*)^, where*f*(*α*) is a scaling exponent of the cells with common *α*. Parameter *f*(*α*) can be calculated as

(6) $$\begin{array}{c} f\left(\alpha \right)=q\alpha \left(q\right)-\tau \left(q\right). \end{array}$$

Multifractal spectrum (MFS), that is, a graph of *α* versus *f*(*α*), quantitatively characterizes variability of the measure studied with asymmetry to the right and left indicating domination of small and large values, respectively. The width of the MF spectrum indicates overall variability [23, 49].

Schertzer and Lovejoy [7, 50] proposed a multifractal model based on the codimension *c*(*γ*). In this model, the scale ratio *λ*  (*λ* = *n*/*δ*) is used instead of *δ* itself being *n* the maximum length size considered (in this case is 1024 pixels). The measure or field (*μ*_*λ*_) is characterized by its probability distribution or by the corresponding law for statistical moments [50]:

(7) $$\begin{array}{c} \text{Pr}\left(\mu_{\lambda }\geq \lambda^{\gamma }\right)\propto \lambda^{-C(\gamma )},\\ \langle \mu_{\lambda }^{q}\rangle \propto \lambda^{K(q)}, \end{array}$$

where 〈 〉 represents the mathematical expectation of the statistical moment, *c*(*γ*) is termed the codimension of a subset with field order greater than *γ*, and *K*(*q*) is the moment scaling function. The relations between *K*(*q*),  *c*(*γ*), and *γ* were derived as [7]

(8) $$\begin{array}{c} K\left(q\right)=\underset{\gamma }{\max}\left(q\gamma -c\left(\gamma \right)\right),\\ c\left(\gamma \right)=\underset{q}{\max}\left(q\gamma -K\left(q\right)\right). \end{array}$$

The characteristics of both functions have been discussed in detail by Schertzer and Lovejoy [7] who proposed a universal model for fitting *c*(*γ*) based on three parameters: *H*, *C*_1_, and *A*. From a statistical point of view, *H* defines the scaling on the mean field, *C*_1_ measures the mean homogeneity of the field or measure the sparseness of the field, and *A* expresses the deviation from the mean of the field values or the “degree” of multifractality.

In the case that *H* = 0 the case studied is a conservative multifractal field, otherwise (*H* > 0) is not and then the analysis applying (4)–(6) to the original measure is insensitive to all the singularities below a critical value so that the ranges of *α* and *f*(*α*) are highly restricted.

In addition, the relationships between their model and the multifractal formalism based on *τ*(*q*), *α*, and *f*(*α*) are the following equations [43]:

(9a) $$\begin{array}{c} \tau \left(q\right)=\left(q-1\right)E-K\left(q\right), \end{array}$$

or

(9b) $$\begin{array}{c} K\left(q\right)=\left(q-1\right)E-\tau \left(q\right), \end{array}$$

(10) $$\begin{array}{c} f\left(\alpha \right)=E-c\left(\gamma \right),\\ \alpha =E-\gamma , \end{array}$$

where *E* is the Euclidean dimension where the measure is embedded.

According to numerous analyses of remote sensing images, the value of *H* is typically around 0.1-0.2 depending on the site and resolution [16, 29, 30, 33, 40, 43]. If 0 < *H* < 1, then taking the absolute gradients (*μ*), instead of the original measure (*m*) of the field, is enough to be able to calculate the full range of singularities. It is therefore important to estimate *H*; in order to do this, a structure function method has been used [51], and based on a bilog plot of the correlation function (*M*_2_(*δ*)) and *δ*, this value was obtained as follows:

(11) $$\begin{array}{c} M_{2}\left(\delta \right)\equiv \langle {\left|\Delta m_{\delta }\left(x,y\right)\right|}^{2}\rangle , \end{array}$$

(12) |Δmδ(x,y)| ≈|m(x,y)    −((m(x+δ,y)+m(x,y+δ)      + m(x−δ,y)+m(x,y−δ))×(4)−1)|,

where *m* refers to the original height value at the point (*x*, *y*) in the DEM.

Then, the original measure was replaced by *μ*(*x*, *y*) = |Δ*m*_1_(*x*, *y*)|  and, based on this, absolute gradient Hölder exponents and MFS were calculated for each case based on (4)–(6), then *K*(*q*) was estimated based on (9b).

## 3. Results and Discussion

### 3.1. Fractal Dimension at Different Threshold Height

**Figure 2:**
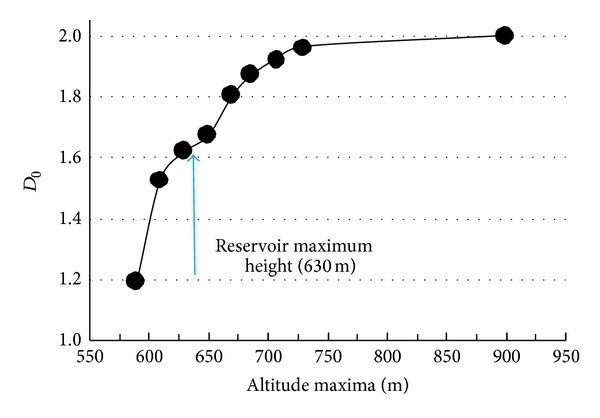
Box-counting dimension (*D*_0_) including points with an altitude less or equal to *x*-axis value with the water reservoir empty. The blue arrow points out *D*_0_ value when the reservoir is full.

First, a preliminary fractal analysis was performed to study how a change in the altitude threshold would affect the fractal dimension (*D*_0_). The intuitive notion of the *D*_0_  of a set of points is that the number of disjoint boxes of size *δ*  (*N*(*δ*)) needed to completely cover the set varies according to (1). Several altitude thresholds (altitude maxima) were applied to DEM data to extract the *D*_0_ of the set of points with an altitude equal or less than a certain value. An increasing *D*_0_ function was obtained by increasing the altitude maximum (see Figure 2).

As the threshold increased, the value of *D*_0_ approached 2 as expected (see Figure 2). However, the function describing this tendency exhibits an inflection point if the maximum altitude considered is the height of the reservoir at its maximum capacity. As the threshold value increases from 630 m to 675 m, the spatial distribution of altitude in the area presents a different pattern from that observed for lower threshold values. The pattern continues to change with further increases in the threshold until 700 m is used as the maximum altitude.

### 3.2. Multifractal Spectrum of the Altitudes

**Figure 3:**
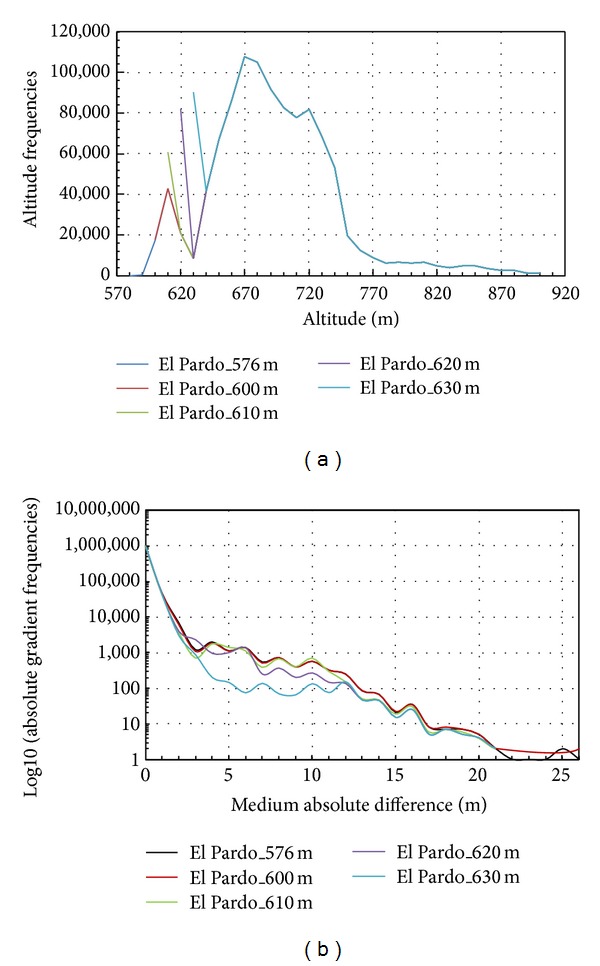
Frequencies distribution, with the water reservoir at different filling levels of (a) altitudes and (b) absolute gradient (frequency in logarithmic scale).

The altitude frequencies for different water levels of the reservoir are shown in Figure 3(a). The only difference among these frequency distributions is the pattern of the lower values. As the water level increases, the minimum altitude increases along with its frequency. We will apply an MFA to each case in which the frequency and the position of the altitude values have a quantitative influence.

**Figure 4:**
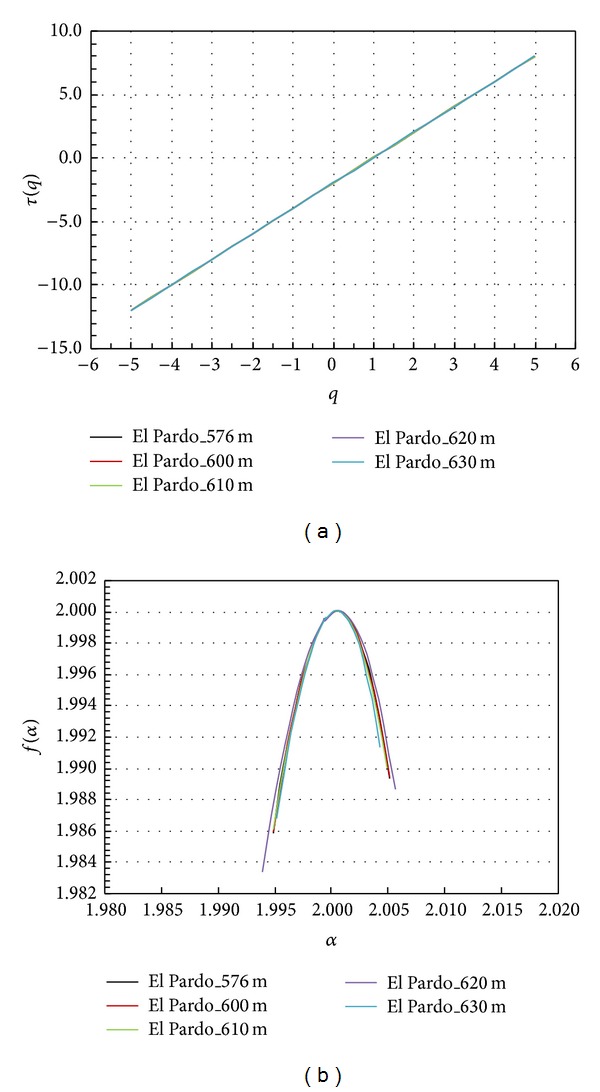
(a) Mass exponent function (*τ*(*q*) versus *q*) and (b) multifractal spectrum (*f*(*α*) versus *α*) based on the original measure (altitude). Each colour represents the water reservoir at different filling levels.

The original measure (altitude) was analysed by first calculating the mass exponent function (*τ*(*q*)) for reservoir water levels of 576, 600, 610, 620, and 630 m. All of them show highly similar *τ*(*q*) behaviour, with a high degree of linearity expressing a low multifractal tendency (Figure 4(a)). The null value for *τ*  (*q* = 1) confirms the conservative character of the measure.

The MF spectra for the five water levels analysed show that the differences among the five spectra with respect to altitude and frequency amplitude are almost null (see Table 1). The value of the Hölder exponent at the box dimension (*α*_0_) is approximately 2 and *α*_1_ is 1.999 and constant in all cases. In contrast, the differences between*f*(*α*_min⁡_) and *f*(*α*_max⁡_) are approximately +0.004, indicating a stronger scaling at high values than at low values, with very tight symmetry in the spectrum (see Figure 4(b)). As the water level of the reservoir increases from 576 m to 610 m, the multifractal parameters are very similar (Table 1), changing slightly for water levels of 610 m and 620 m.

### 3.3. Multifractal Spectrum of the Absolute Gradient

**Figure 5:**
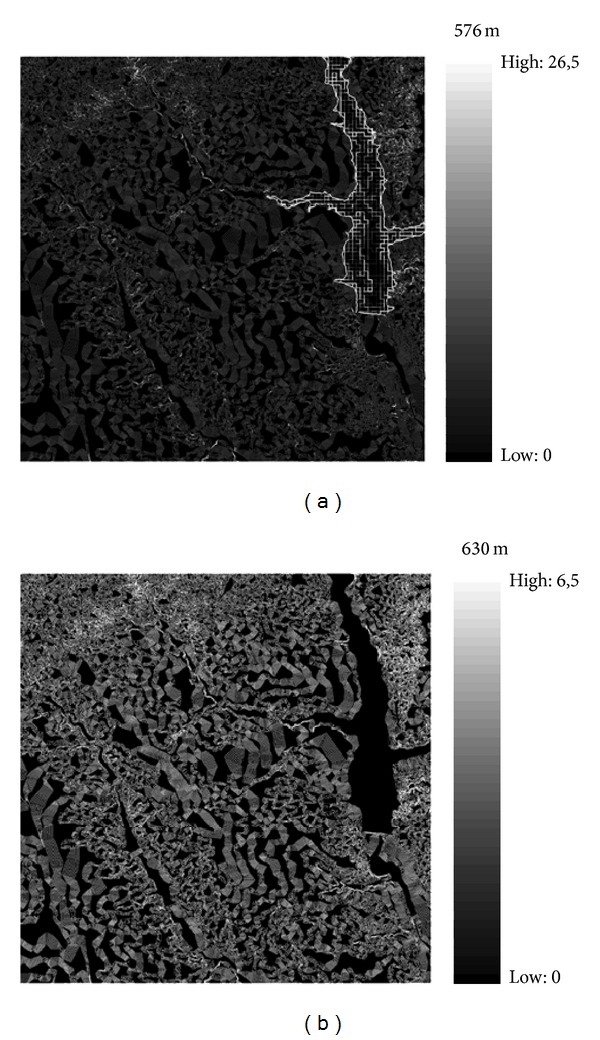
Absolute gradient, |Δ*m*_*δ*_(*x*, *y*)| ≈ |*m*(*x*, *y*)−(*m*(*x* + *δ*, *y*) + *m*(*x*, *y* + *δ*) + *m*(*x* − *δ*, *y*) + *m*(*x*, *y* − *δ*))/4|, where *m* refers to the original altitude value at the point (*x*, *y*) in DEM and *δ* = 1. (a) With the water reservoir empty (minimum altitude 576 m). (b) With the water reservoir full (minimum altitude 630 m).

The same type of analysis was applied after the data were transformed (see (12)) to an absolute gradient. The results of this transformation for the cases of an empty reservoir and a reservoir at maximum capacity are illustrated in Figures 5(a) and 5(b). A comparison of this figure with Figure 1(a) highlights the differences between the results of the analysis for the original measure and the transformed data. In the analysis of the absolute gradient, the points showing the greatest differences from the points surrounding them (edges) are the higher values. In contrast, the lower values of the absolute gradient show almost no differences from the surrounding points (darker colour in Figure 5).

These differences are even more pronounced if the frequencies of the absolute gradient are plotted for each case study (see Figure 3(b)). The distributions for the different case studies are similar. However, the cases considered show a pattern as the water level increases. As the water level of the reservoir increases to 630 m, the distribution becomes steepest. This tendency is a result of the increase in the area of the reservoir as the reservoir is filled. This process increases the frequency of 0 and 1 values of the absolute gradient. When the water level of the reservoir is 630 m, the distribution shows its greatest slope for absolute gradient values less than or equal to 5 m. The frequencies are lower ranging from 5 to 11 m. For values greater than 11 m, the behaviour for the water level of 630 m is similar to that for the other water levels. Although the differences shown in Figure 3(b) appear to be minimal, they have implications for the MFA, as we will show below.

**Figure 6:**
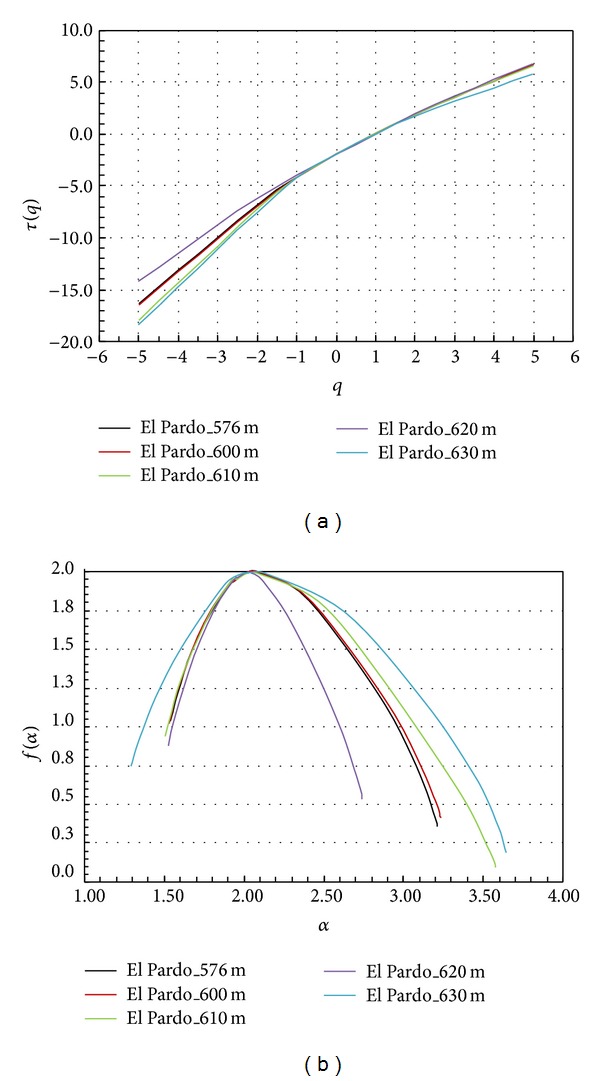
(a) Mass exponent function (*τ*(*q*) versus *q*) and (b) multifractal spectrum (*f*(*α*) versus *α*) based on absolute gradient. Each colour represents the water reservoir at different filling levels.

The nonlinearity observed in *τ*(*q*) (Figure 6(a)) implies a scale dependence of the dimensionless moments and, therefore, a pronounced MF character versus the behaviour shown in the MFA of the original measure (Figure 4(a)). This richness in multiscaling behaviour is shown in Figure 5. The spatial distribution of the mean absolute gradient displays a more complex pattern, highlighting the points with a greater number of rough edges. At the next step, the MF spectrum shows different amplitudes for the different water levels of the reservoir (Figure 6(b)). This behaviour is clear from Table 2.

The value of the Hölder exponent at the box dimension (*α*_0_) is slightly greater than 2. *α*_1_ ranges from 1.93 to 1.98, with a tendency to increase as the water level increases. The values of altitude and frequency amplitude for the five cases studied are higher and more significant than in the MF analysis of the original measure (compare Tables 1 and 2). In general, Δ*α* and Δ*f* increase with the filling of the reservoir. However, there are exceptions to this pattern. At El Pardo_620, Δ*α* shows a decrease to 1.218, and there is a singular value of −0.840 at El Pardo_610.

The general increase in Δ*α* implies an increase in overall variability in space. As it is clear from Figure 5, the highest values are concentrated around the limits of the reservoir when the reservoir is empty. This tendency no longer holds when the reservoir is full, as a more complex structure with higher spatial variability develops. The differences in *f*(*α*_min⁡_) and *f*(*α*_max⁡_) are all negative, indicating a stronger scaling at low values than at high values, with no pattern of symmetry in the spectrum (see Figure 6(b)).

**Figure 7:**
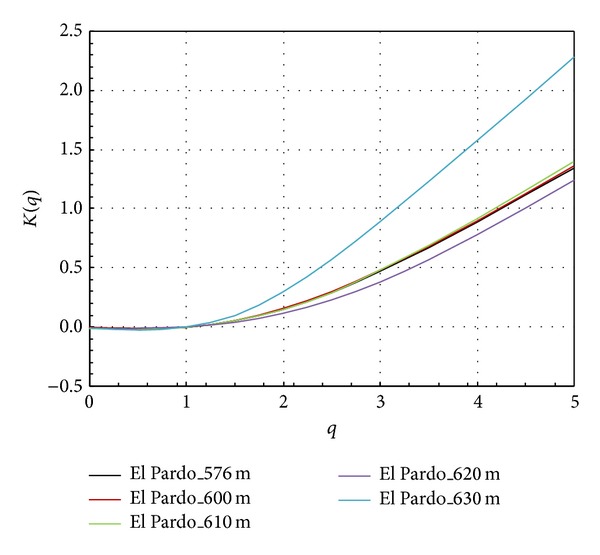
Moment scaling function (*k*(*q*)  versus  *q*) based on the absolute gradient. Each colour represents the water reservoir at different filling levels.

If we transform the multifractal spectrum into a moment scaling function (*K*(*q*)), we obtain a clear picture of the difference between the case of the full reservoir (El Pardo_630) and the other water levels (see Figure 7). In all of the cases studied, the moment scaling functions are the same for *q* ≤ 1. The differences are found for *q* > 1.

## 4. Conclusions

The goal of this study was to examine the multiscale statistical properties of the altitude and the absolute gradient in an area of homogeneous soil. In this area, the topography and the reservoir constructed on the river played a main role. Such characterisation is related to the spatial organisation of the landscape and could shed light on its evolution.

Several clear results have emerged from this analysis. First, topographic altitude exhibits a weak multiscale statistical structure and a negligible deviation from scale invariance or monoscaling when a multifractal spectrum is obtained. Second, if the original measure (altitude) is replaced by the mean absolute gradient (or mean absolute difference), the multiscale analysis reveals a higher degree of multifractality, allowing a more informative analysis of the influence of the water level of the reservoir.

By addressing the issues of structure and scale, the multifractal formalism, unlike classical geomorphometrical tools, provides scale-invariant attributes for characterising topography and landscapes. The results of this study show that the use of the multifractal approach with mean absolute gradient data is a useful tool for analysing the topography represented by the digital elevation model.

## Figures and Tables

**Table 1:** Parameters extracted from the multifractal spectrum based on the original measure (altitude) with the water reservoir at different filling levels. Holder exponent at *q* = −5  (*α*_max⁡_), = 0 (*α*_0_), *q* = 1 (*α*_1_), *q* = +5 (*α*_min⁡_), and *α*_max⁡_ − *α*_min⁡_ (Δ*α*). Multifractal value at *α*_max⁡_(*f*(*α*_max⁡_)), *α*_min⁡_(*f*(*α*_min⁡_)), and *f*(*α*_max⁡_) − *f*(*α*_min⁡_)(Δ*f*).

	*α*_min⁡_	*α*_0_	*α*_1_	*α*_max _	Δ*α*	*f*(*α*_min⁡_)	*f*(*α*_max⁡_)	Δ*f*
El Pardo_576	1.995	2.001	1.999	2.005	0.010	1.986	1.989	0.003
El Pardo_600	1.995	2.001	1.999	2.005	0.010	1.986	1.989	0.003
El Pardo_610	1.995	2.000	1.999	2.005	0.010	1.986	1.990	0.004
El Pardo_620	1.994	2.001	1.999	2.006	0.012	1.983	1.989	0.006
El Pardo_630	1.995	2.001	2.000	2.004	0.009	1.987	1.991	0.004

**Table 2:** Parameters extracted from the multifractal spectrum based on the average absolute differences of altitudes with the water reservoir at different filling levels. Holder exponent at *q* = −5  (*α*_max⁡_), *q* = 0 (*α*_0_), *q* = 1 (*α*_1_), *q* = +5 (*α*_min⁡_), and *α*_max⁡_ − *α*_min⁡_ (Δ*α*). Multifractal value at *α*_max⁡_(*f*(*α*_max⁡_)), *α*_min⁡_(*f*(*α*_min⁡_)), and *f*(*α*_max⁡_) − *f*(*α*_min⁡_)(Δ*f*).

	*α*_min⁡_	*α*_0_	*α*_1_	*α*_max _	Δ*α*	*f*(*α*_min⁡_)	*f*(*α*_max⁡_)	Δ*f*
El Pardo_576	1,539	2,094	1,930	3,213	1,674	1,033	0,357	−0,676
El Pardo_600	1,539	2,098	1,932	3,226	1,687	1,033	0,409	−0,624
El Pardo_610	1,509	2,092	1,950	3,580	2,071	0,098	0,938	−0,840
El Pardo_620	1,527	2,045	1,960	2,745	1,218	0,873	0,528	−0,345
El Pardo_630	1,294	2,124	1,980	3,645	2,351	0,744	0,186	−0,558
